# Sequences in popular cinema generate inconsistent event segmentation

**DOI:** 10.3758/s13414-019-01757-w

**Published:** 2019-05-15

**Authors:** James E. Cutting

**Affiliations:** Department of Psychology, Cornell University, Ithaca, NY USA

**Keywords:** Continuity, Events, Movies, Narrative, Scene, Segmentation, Sequence, Soundtrack

## Abstract

Popular movies have an event structure that includes scenes and sequences. Scenes are fashioned to be perceived as smoothly flowing, a feature called *continuity*. Discontinuity is said to occur when scene (event) boundaries are crossed. This article focuses on the structure and perception of sequences that have subscenes (i.e., scene-like components) but whose boundaries, unlike those of scenes, tend to demonstrate some perceived continuity. Although the structure of sequences has been addressed by film theory, this topic has not received psychological attention. Here, data are used from viewer judgments and physical measurements of 24 popular movies, released from 1940 to 2010. Each film was inspected for narrative shift patterns—that is, changes in location, character, or time—across shots. Sequences were determined by repeated shift types, common sound coverage, and the shorter durations of subscenes than of scenes. By these criteria, sequences have increased in movies over time. The results also show that viewer judgments of event boundaries diminish in the presence of music and of shorter and less modulated shot durations. These results fit snugly within event segmentation theory, and this categorization of movie sequences by narrative shifts can accommodate previous accounts of sequence structure.

## Movie scenes are events

“Life is just one thing after another” (Keillor, 2013, 8:6; Radvansky, 2012, p. 269), and a popular movie is, too. Importantly, the “things” that compose our experiences in life are called *events*, and those that compose movies are called *scenes*. Real-world events and movie scenes have something of the same structure. Generalizing from Aristotle, they both typically have beginnings, middles, and ends. This is not a vacuous claim: Beginnings and ends of events in life tend to have more motion or change than their middles (Zacks, Swallow, Vettel, & McAvoy, 2006), and beginnings and ends of movie scenes have different shot durations and scales than their middles (Cutting, Brunick, & Candan, 2012). Moreover, many agree that “in a well-edited movie, cuts that classical film theory would identify as scene breaks should be identified as event boundaries” (Zacks & Magliano, 2011, p. 442; see also Cutting, 2014; Tan, 2018; Zacks, 2015).

The boundaries of real-world events and of scenes are particularly important. What follows an end is less predictable than what follows a middle (Zacks & Swallow, 2007), and a new beginning initiates the formation of a new schema for understanding—an event model (Zacks, Speer, Swallow, Braver, & Reynolds, 2007), analogous to a situation model in prose understanding (Zwaan & Radvansky, 1998). Moreover, in both purpose-made research videos and edited professional films, event boundaries can affect memory, in that boundaries that intercede between an item to be remembered and its test can impede its recall (Swallow, Zacks, & Abrams, 2009).

Scenes in popular movies are designed in part to speed up the narrative. As Alfred Hitchcock noted, “What is drama, after all, but life with the dulls bits cut out” (Truffaut & Scott, 1983, p. 103). Scene boundaries are important demarcations where a number of attributes of the narration can change. According to event segmentation theory (Zacks et al., 2007; Zacks & Swallow, 2007), the event perceiver observes the ongoing activity and tacitly makes predictions about what will happen next. When the predictions and happenings mismatch, an error signal occurs in the mind of the perceiver. This is a sign that one event has ended, another has begun, and that she should close off one event model and create another. Together, such models serve as integrated and interconnected memory schemata, analogous to Gernsbacher’s (1995) structure-building framework for language comprehension.

In cinematic terms, one task of the filmmaker is to make sure that false predictions do not typically occur within a scene, but that they can occur at scene boundaries. The means of eliminating within-scene unpredictability is called *continuity editing*. The task of signaling an event change in cinema is done through the discontinuity resulting from changed locations, characters, and/or time frames—which my students and I have called *narrative shifts* (Cutting, 2014; Cutting et al., 2012). Below I will consider these in more detail.

## Continuity

Continuity is a central goal of popular filmmaking. It is the flow that drives a narrative forward. But the filmmakers’ task is more difficult than it may first appear. Popular contemporary movies have as many as two or three thousand cuts, or abrupt changes in the visual stream, that occur before and after each shot. Only about 10% of these are scene boundaries. Thus, cuts are ambiguous information—most shots *are not* events, but most scenes *are* events (Cutting, 2014; Magliano & Zacks, 2011), yet cuts typically form the beginnings and endings of both.

Popular filmmakers try to achieve continuity by adhering to a loose toolkit of techniques discovered over more than a century. That toolkit includes rules allied with many psychologically relevant principles (Berliner & Cohen, 2011; Smith, 2012). Several mimic how one physically joins a group in the real world: (a) Scenes are introduced with an establishing shot that shows the environment and spatial locations of the characters at some distance; then (b) the camera moves in for medium shots and medium close-ups, typically (c) alternating views of characters while staying on the same side of an axis that goes between them (the 180° rule).

Other techniques concern low-level perceptual phenomena: avoiding apparent motion at cuts between shots by (d) moving the camera sufficiently to the side between shots (the 30° rule), and capitalizing on continuous directional motion (Ball & Sekuler, 1979) by (e) matching on action across shots (the direction of motion at the end of one shot matching that at the beginning of the next). Yet another technique is to have the character in view and the camera mimic shared attention (Moore & Dunham, 1995) by (f) matching the eye line (or sight line) across shots. That is, when a character looks off screen, the next shot shows what she looked at, from the general screen height and location of the character’s eyes.

Conceptual groundwork is also laid for the narrative future, (g) motivating the switch to a new scene or the context of a later one by information presented at a scene’s close (a “dangling cause”; see Thompson, 1999), while “hooking” one scene to the next by graphic matches, action matches, sound bridges, or audiovisual linkages (Bordwell, 2008). Moreover, this short summary does not exhaust the list (see Thompson, 1985).

Continuity and discontinuity have been experimentally assessed using at least four psychological techniques. The first is the detection of cuts (abrupt visual changes) in the ongoing film stream (Smith & Henderson, 2008; Smith & Santacreu, 2017), where lower accuracy can be taken as evidence of continuity, and higher accuracy for discontinuity. Indeed, cuts are easier to detect at scene boundaries. A second technique is the measurement of eye movement activity across cuts (Smith & Santacreu, 2017; Swenberg & Eriksson, 2017), where the greater the disruption, the greater the discontinuity. Digital manipulation of the ends of shots can increase eye movements and cut detection. Third, as was noted above, psychologists have assessed memory for details that come before versus after an event (scene or scene-like) boundary (Swallow et al., 2009), where decreased performance implies discontinuity. And finally, observers may segment a video stream into parts (Cutting et al., 2012; Zacks & Swallow, 2007), where ignoring certain potential boundaries can be taken as evidence for continuity. This article pursues evidence using this fourth method.

## Codes for narrative shifts

Theater practice (Polking, 1990) suggests that scenes are separated by changes in one or more of three attributes—location, characters, or time. My students and I have called them *narrative shifts* (Cutting, 2014; Cutting et al., 2012). In movies, these discontinuities occur across a pair of shots that end one scene and begin the next. There are seven types of such shifts, which can be given a three-place code: [L – –] for a change in location, [– C –] for a change in main characters, and [– – T] for a change in time frame. Thus, the seven types are [L C T], [L C –], [L – T], [L – –], [– C T], [– C –], and [– – T], with the eighth type [– – –] representing no shift, or the within-scene juxtaposition of shots.^1^ This nomenclature will be useful later, so to help keep these in mind, they are listed and described in Table 1.

**Table 1:** Narrative shifts, their codes, and their relative frequencies in whole movies, in scenes, and in sequences in a sample of 24 films released from 1940 to 2010

Code	Description	Percentage of Narrative Shifts Overall	Percentage of Narrative Shifts Excluding Sequences	Percentage of Narrative Shifts Within Sequences
[L C T]	Shifts in location, characters, and time	30.3	37.4	20.6
[L C –]	Shifts in location and characters, but not time	40.3	35.2	47.1
[L – T]	Shifts in location and time, but not characters	6.9	0.9	15.5
[L – –]	Shift in location, but not characters or time	7.6	10.0	4.4
[– C T]	Shifts in characters and time, but not location	0.5	0.1	1.2
[– C –]	Shift in characters, but not in location or time	13.3	16.1	9.6
[– – T]	Shift in time, but not in characters or location	1.1	0.5	2.0

**Fig. 1:**
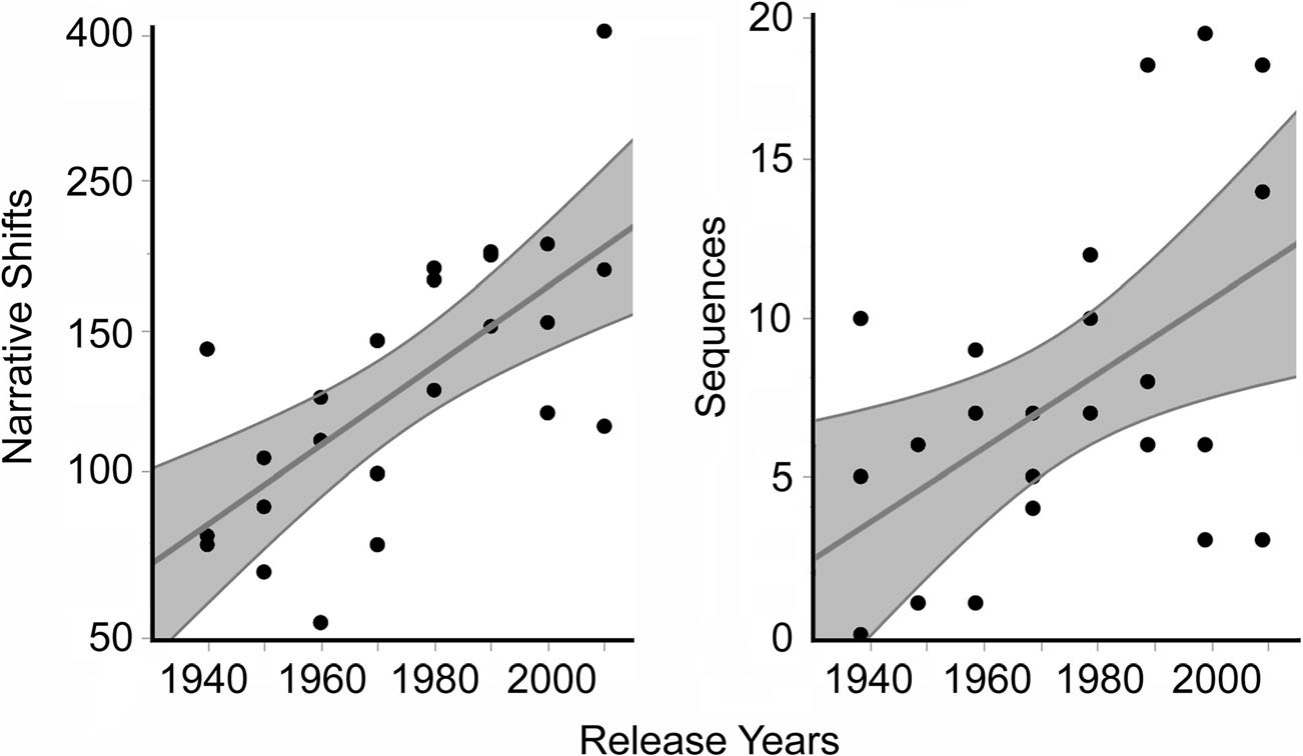
The left panel shows the numbers of narrative shifts across 24 films (black dots) released from 1940 to 2010 (adapted from Cutting, 2019). The right panel shows the numbers of sequences in those same movies from Study 1. The areas shaded in lighter gray show the 95% confidence intervals on the regression lines (in darker gray)

Consider the frequency data on these narrative shifts. Cutting et al. (2012) coded the narrative shifts of 24 popular films released between 1940 and 2010 (see their appendix for the list of movies). The records of the narrative shifts in these movies were updated by Cutting (2014), with a few additional shifts included, and again for this article, where a few more can be found, for a current total of 3,100. Their overall relative frequencies are given in the first data column of Table 1. Notice that two shift types are quite common: [L C T], in which all attributes change across a scene boundary, and [L C –], in which time does not change but the location and characters do. These two types make up 70% of all narrative shifts in this sample of movies. Three other types are relatively uncommon—[L – T], [L – –], and [– C –]—and two are quite rare—[– C T] and [– – T]. Across movies, narrative shifts have increased in their number from 1940 to 2010, as is shown in the left panel of Fig. 1 (*r* = .66), *t*(22) = 4.38, *p* = .0002, *d* = 1.86. This increase seems to signal a growth in narrational complexity in popular movies over the last 70 years, or perhaps even longer (Cutting, 2019).

## Movie sequences

The goals of this article is to propose a new structure for sequences, to gather empirical data in support of this proposal, and to tie both to a previous account of sequences and to event segmentation theory. A *sequence* is a structure made of up scene-like units—or *subscenes* (Cutting, 2014; Cutting et al., 2012). Bellour (1976) called them *subsegments*. The problem is that these units, unlike scenes, don’t seem to have beginnings and ends. Instead, many seem more like ongoing middles, often crosscut between two or more narrative threads. Among the more obvious examples in popular movies, sequences include (a) the high-climax sections of action films, in which shot groupings of the protagonist and of the antagonist alternate until a final confrontation is joined; (b) battles, in which different combatants are sequentially picked out of a crowded chaos; and (c) chases, in which the camera position changes locations alternately depicting the chaser and the chased.

## Defining a sequence

### Three criteria

Here I consider an aggregation of consecutive (sub)scenes as a sequence if it meets three criteria. First, it must have a repetition pattern of narrative shift codes. That is, it needs at least two successive narrative-shift boundaries of the same type—for example, [L C –]:[L C –]. The two-shift minimum entails at least three successive subscenes.

Second, these three or more subscenes are bound in the soundtrack. This binding creates a “sonic flow” (Chion, 1994, pp. 54ff). Sequences are covered by music (either diegetic or nondiegetic, where the former would be heard by the characters in the movie, and the latter would not), by voiceover, or by a pervasive auditory signal.

Third, each of the successive subscenes is shorter than the mean scene duration of that particular movie. This last criterion is invoked for two reasons: (a) sequences generally have a faster pace, and their subscenes generally have fewer shots than the scenes of the rest of the movie, and (b) without this stipulation, many contemporary action movies could be considered as essentially one long set of sequences.

### Additional assumptions

Several other decision rules are important for these categorizations, as was established by the author-coders in Cutting et al. (2012). Locations change when boundaries are crossed. The most obvious boundaries are doors (Radvansky & Copeland, 2006). Outdoors, on the other hand, boundaries occur with geographical differences—playing fields versus spectator stands, one side of a river as opposed to the other, or changes in terrain or plant life. If the location changes significantly across shots with the same characters shown, time necessarily changes as well—unless there is some kind of instantaneous transport, as in *Star Trek: The Motion Picture* (Wise, 1979) or in *Jumper* (*Liman, 2008).^2^

Second, character shifts are noted only for major characters, and each movie typically has between six and eight of them. Here, a major character is defined as one appearing in more than 10% of the movie’s shots. When no major characters are in a shot and the next shot also has no major characters, the character code is null. When scene changes go from no main characters to characters present, or the reverse, the character code is [– C –]. And when a main character enters or exits a location already having characters, this too is a character change.

Third, concerning time, I assume that at cross-cuts of narrative threads, time runs continuously. That is, time does not back up or skip ahead unless it is somehow explained with a dissolve, day changing to night, or other transition.^3^

## Elaborations

### Montages

A number of shorter sequences fall into a special category often called the “Hollywood montage” (Cutting, Brunick, & DeLong, 2011; Dmytryk, 1984; Salt, 2009). These entail successive shots traditionally separated by dissolves. These shots are thematically related but often occur in different locations, with different characters, and/or at different times. Consider some examples.

Some montages show travels in space across time, where the same characters are seen on a trek, voyage, or trip: [L – T]. For example, one occurs in the *Grapes of Wrath* (*Ford, 1940), showing the Joad family moving west, with overlays showing signs of the towns they pass through. A similar one can be seen in *Detour* (*Ulmer, 1945), as the protagonist hitchhikes across the country. Others show travels in time, where the same characters are followed across time but remain in the same general location. An example of this [– – T] type is found in *The Blue Lagoon* (*Kleiser, 1980), in which marooned children grow older as they explore their island under the guidance of the one adult who survived their shipwreck. In addition, there are prologue montages, which introduce a movie (often with superimposed credits) and often have no important characters but contain video snippets that portray various locations in the movie’s setting with no commitment to time frame, [L – –]. An example with dissolves occurs at the beginning of *Ordinary People* (*Redford, 1980), and one without dissolves occurs at the beginning of *The Sound of Music* (*Wise, 1965).

In general, montages with dissolves have been less common since about 1980 (Cutting et al., 2011). In more contemporary movies, the single consecutive shots are simply separated by a cut—like most of the shots in the rest of the movie. Another montage without dissolves occurs with the Layla sequence in *Goodfellas* (*Scorsese, 1990)—[L C –]—in which mobsters show up dead in cars, trash skips, and meat trucks. Because of the pulsing music coverage and voiceover, we interpret this series of events as matters of fact in the mob world, not a sequence of tragedies.

### Simple and complex sequences

Finally, there are two general classes of sequences—simple, which I focus on in this article, and complex. Following the scheme above, a simple sequence is the successive concatenation of the same type of narrative shift. Importantly, all of these simple sequences are preceded and followed by normal scenes, and they comprise 78% of this sample.

The remaining 22% are complex sequences. Some of these are actually simple sequences abutted end to end. An example can be found in the opening shots of *The Perfect Storm* (*Peterson, 2000). This movie begins with a montage of five shots around the Gloucester harbor, [L – –], but then switches to the interior of the town hall and a four-shot montage of a wall showing the dates of fishermen who died at sea. The latter can be described as a [– – –] sequence, in which short drifting glimpses of names on the various sections of the wall are shown. The same nondiegetic music covers both.

Other complex sequences are like meta-sequences, or sequences of sequences. These typically maintain the standard repetition for a while, but then vary it even while it is covered by the same music. One example can be found in the climactic, 7-min motorcycle chase sequence in *Mission: Impossible II* (*Woo, 2000), in which Ethan Hunt’s (Tom Cruise’s) escape and chase-down by his antagonist are intercut with subscenes of Hunt’s crew in a helicopter, the antagonist’s crew in cars, various side battles, and Hunt’s girlfriend wandering while infected with a plague-like virus. Most of this could be coded as [– C –] and [L C –]—with changes in characters through continuous time, and some changes in locations—but the interweaving of five narrative threads takes it beyond what I wish to consider here.

With this background in place, it is time to consider data. The exposition here entails four studies. The first assesses the distribution of sequences categorized by narrative-shift types, for the purpose of comparing them with the narrative shifts between scenes. The second looks at viewer segmentations of subscenes within sequences, as compared to those between scenes. The third looks at the shapes of shot durations and shot scales in subscenes, as compared to those of scenes. Finally, the fourth assesses the relative importance of music, shot duration, shot scale, motion, luminance, and clutter in viewers’ segmentations of the movie stream.

## Study 1: Distribution and examples of simple sequence types

### Method

Across a sample of 24 movies—three movies (an action film, a comedy, and a drama) from each of eight years (1940, 1950, 1960, 1970, 1980, 1990, 2000, and 2010), all among the most popular films of their release year (Cutting, 2014; Cutting et al., 2012)—my students and I coded as many narrative shifts as we could determine. From our earlier records, I scanned for all occurrences of the same narrative shift type that occurred successively at least twice, and entailing subscenes with at least three shots each. I then rewatched those candidate sequences in each movie many times to determine whether they met the three criteria, and then categorized them.

### Results

The total number of sequences per movie increases with release years, as is shown in the right panel of Fig. 1 (*r* = .47), *t*(22) = 2.76, *p* = .012, *d* = 1.17. This trend is correlated with the numbers of narrative shifts, in the left panel (*r* = .75), *t*(22) = 5.3, *p* < .0001, *d* = 2.27. Four of the original set of movies had one sequence or none (one in 1940, two in 1950, and one in 1960), and were omitted from further consideration. This left 20 movies in the sample.

Following my guidelines, I found 180 sequences. Of these, 81% were accompanied by nondiegetic music, 10% by diegetic music, 5% by voiceovers, and 4% by simpler audio continuity. The distribution of narrative shifts in these sequences is given in Table 1, along with the distribution of shifts among the scenes outside of sequences. The correlation of frequencies for narrative shifts between scenes against those between subscenes within sequences is reasonably strong (*r* = .77), *t*(5) = 2.7, *p* = .021. Most striking, however, is that almost all of the [L – T] shifts occur within sequences in which a character sequentially changes several locations over time.

The most common simple sequence type occurs with a string of [L C –] transitions, in which locations and characters alternate but time runs continuously. Indeed, whole action movies can be composed of [L C –] alternations with few other shifts, such as *Die Hard 2* (Harlin, 1990). These represent just less than half (47%) of all simple sequences in this sample. A compelling 3-min example of this type occurs in the hacking sequence after the opening credits in *The Social Network* (*Fincher, 2010). It alternates 15 times between dorm-room computer coding by Mark Zuckerberg (Jesse Eisenberg) and partying at Harvard social clubs. Nondiegetic music occurs throughout, with music throbbing during the party subscenes and music more subtly accompanying Zuckerberg’s voiceovers in the dorm room.

The second most common simple sequence occurs with a string of [L C T] transitions (21%), in which all dimensions change across subscenes. One 2-min sequence of location, character, and time changes occurs at the climax of *Ordinary People* (*Redford, 1980), in which high-schooler Conrad Jarrett (Timothy Hutton) discovers that his friend from the hospital, Karen, has killed herself. The subscenes of this discovery—his trip to the bathroom to consider slitting his wrists again, and his eventual run to the therapist’s office—are interspersed with quick, blue-tinted flashbacks of the boating accident that he survived but that drowned his brother. The sequence is covered with slow but continuous piano music that accelerates in orchestral breadth.

Third, and only slightly less frequent (16%), are the sequences consisting of a string of [L – T] transitions. A salient case is a 30-s sequence, also from *Ordinary People*, in which location and time change but the characters do not. Calvin Jarrett (Donald Sutherland), father of Conrad, has a reverie about good times with his wife, Beth (Mary Tyler Moore). The shots alternate between their stony silence aboard a plane and them dancing gloriously at a previous time. Diegetic despair is apparent in part because the dancing is not accompanied by music, but instead the whole sequence is covered by the low-pitched drone of the jet engines. Here, as elsewhere in *Ordinary People*, the reverie subscenes are chromatically different from the diegetic subscenes, likely to help the viewer keep the situations and times separate (Bordwell, 1985).

The fourth most common sequence occurs with a string of [– C –] transitions (10%). The longest of these in this sample are two 6-min sequences that occur near the end of *MASH* (*Altman, 1970). Both halves of a football game are shown, as if continuous in time and location, but the characters change very often—showing those on the field, those on both benches, and those on both the sidelines. Marching-band nondiegetic music generally accompanies throughout.

The other three sequence types are rare in this sample (8% total). It is difficult to change locations without changing characters and time: [L – –]. The easiest way to do this is a series of depictions with no characters at all, as in the opening sequences of *Ordinary People* (*Redford, 1980) and *The Perfect Storm* (*Peterson, 2000), both already discussed. One of the three [– – T] sequences in this sample occurs in *Mission: Impossible II* (*Woo, 2000) just before the opening credits, with Ethan Hunt rock climbing, accompanied by sounds of breathing and the wind, and eventually thumping nondiegetic music. Jump cuts (indications of time change) show the same character on the same climb. Finally, there is also only one example of a [– C T] sequence in this sample—the first part of gladiator training in *Spartacus* (*Kubrick, 1960), in which the trainees (Spartacus included but not shown exclusively) go through various tests in the same arena, with skips in time covered by brash brass music. As is shown in Table 1, the [– – T] and [– C T] narrative shifts are very rare generally, and thus one might not expect them to be strung together very often.

### Conclusion

The typology of seven different kinds of simple sequences based on narrative shifts seems effective. Examples can be found of each. Moreover, the general frequency of narrative shifts between subscenes seems to be of the same order as those between scenes, with the exception of narrative shifts of the [L – T] type, which seem almost exclusively confined to sequence transitions between subscenes. But are the subscenes within sequences segmented in the cinematic stream less frequently than scenes?

## Study 2: Scenes are segmented, subscenes are often not

### Method

Cutting et al. (2012) had eight viewers segment the 24 movies from Study 1. Using a modified two-pass procedure (Magliano & Zacks, 2011; Zacks et al., 2006), the researchers had viewers watch entire movies twice, first to simply enjoy and experience the movie, and second to segment them. In the second task, viewers scrolled frame by frame through the movies and noted the beginning frame of each new event. Shot boundaries were chosen almost exclusively. The time taken and tediousness of the second pass was made up for by the accuracy with which we know the event boundary. Viewers agreed to a reasonable degree (mean *κ* = .56). In particular, on 85% of the shots they concurred that no new event had begun; on 8% of the shots, they all noted that a new event had begun; and on only 7% was there disagreement.

For each of the 20 movies that had at least three subscenes, as determined by Study 1, I analyzed the frequency with which narrative shifts were judged by the three viewers as boundaries between scene and subscene segments. The values could be 0, 1, 2, or 3, representing the number of viewers who segmented the movies at a given narrative shift. Across the 3,100 narrative shifts, the mean values converged on a continuous measure.

### Results

**Fig. 2:**
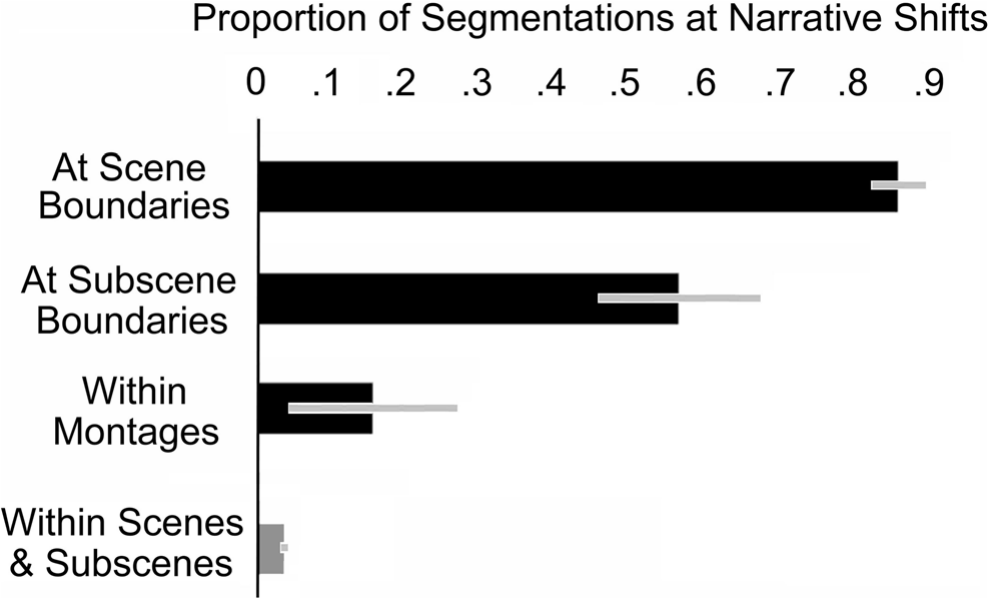
Comparison of viewer segmentation performance at narrative shifts between scenes, between subscenes within sequences (excluding montages), and within montages (bars in black). These values are compared to the within-scene or within-subscene segmentation rate (in gray) without a narrative shift. Error bars indicate 95% confidence intervals

Scenes were reasonably well segmented on the basis of narrative shifts (85%), whereas narrative shifts within sequences were less often noticed (44%), *t*(45.8) = 6.56, *p* < .0001, *d* = 1.94, as is shown in Fig. 2. This difference strongly suggests that viewers think that whole sequences are more likely to be psychological units than their component subscenes. However, this result seemed odd, since the populations of narrative shifts separating scenes and subscenes are nearly the same (except for the [L – T] shifts), as can be seen in Table 1. The conjunction of structural similarity and psychological difference suggests that narrative shifts in sequences are somehow partly masked.

Another large difference is found in the segmentations at narrative shifts within sequences versus those within montages, also shown in Fig. 2. Across the 13 movies that had both multishot subscenes in sequences and single-shot alternations in montages, there was a large difference in the segmentation for subscenes (56%) versus montages (15%), *t*(31.4) = 2.89, *p* = .007, *d* = 1.03. Strikingly, the segmentation of montages is not much different from the rate of segmenting shots within a scene or subscene: 4%, *t*(11) = 2.08, *p* = .06. Montages are perceived essentially as ordinary scenes.

### Conclusion

Viewers competently use narrative shifts to segment movies into scenes, doing so with a response rate of 85% in this sample, a result consistent with Magliano and Zacks (2011). Viewers almost completely ignore narrative shifts between the shots of montages, segmenting at a rate of 15%. Between these extremes is viewers’ consistency in segmenting subscenes within sequences: 56% in films when montages are excluded. If narrative shifts are markers of discontinuity, the less consistent performance on within-sequence transitions of the same kind as between-scene transitions represents a masking of narrative-shift discontinuity. Studies 3 and 4 addressed why this masking might occur.

## Study 3: The different shapes of scenes and subscenes

The central conundrum of this investigation concerned the conjunction of two facts: the similar distributions of narrative shifts employed at scene and subscene boundaries (Table 1), and the difference in viewers’ segmentations at those boundaries (Study 2). One possible solution concerns the nature of the shot durations and shot scales in both cases. Cutting et al. (2012) found that together these accounted for 21% of the variance in viewer segmentations. Perhaps subscenes really don’t have salient beginnings and ends.

### Method

The residual 20 movies were again used. Only subscenes with at least three shots were considered. This stipulation was followed in order to track any nonlinearities in their profile shapes. Each shot vector of each subscene was affine-transformed using the method of Cutting et al. (2012). In particular, the shot values (both scale scales and shot durations) were first interpolated (oversampled) to fit in 200 bins. This was done so that all subscenes could then be averaged within a movie for their overall shape and, more importantly, compared with the values found for the scene profiles of those same 20 movies. Since the values in these 200 bins were not independent, they could not be used for statistical analysis. Thus, the mean data for scenes and subscenes in each movie were taken from seven bins—Bins 1, 33, 67, 100, 133, 167, and 200. These were selected in order to decrease the degrees of freedom in the regression analysis and to align with the median number of shots (7) per subscene. Because of their strongly positive skew, the shot durations were log-scaled for analysis.

### Results

The grand total of subscenes meeting these criteria was 511, with a mean of 25.6 per movie and a median of 15. As had been found by Cutting et al. (2012), there was a reliable effect of release year in the log-transformed analysis [*F*(1, 275) = 153, *p* < .0001], with older movies having longer shot durations. There was also an effect of genre [*F*(2, 275) = 32.4, *p* < .0001], with action movies having shorter durations (4.1 s) than comedies (5.5 s), which in turn had shorter durations than dramas (6.7 s). There were no reliable interactions among these variables. However, a quadratic trend did emerge across the seven bins [*F*(2, 277) = 5.66, *p* = .004, *d* = 0.40]. That is, shot durations were longer at the beginning and end boundaries of scenes and subscenes combined, consistent with Cutting et al. (2012).

**Fig. 3:**
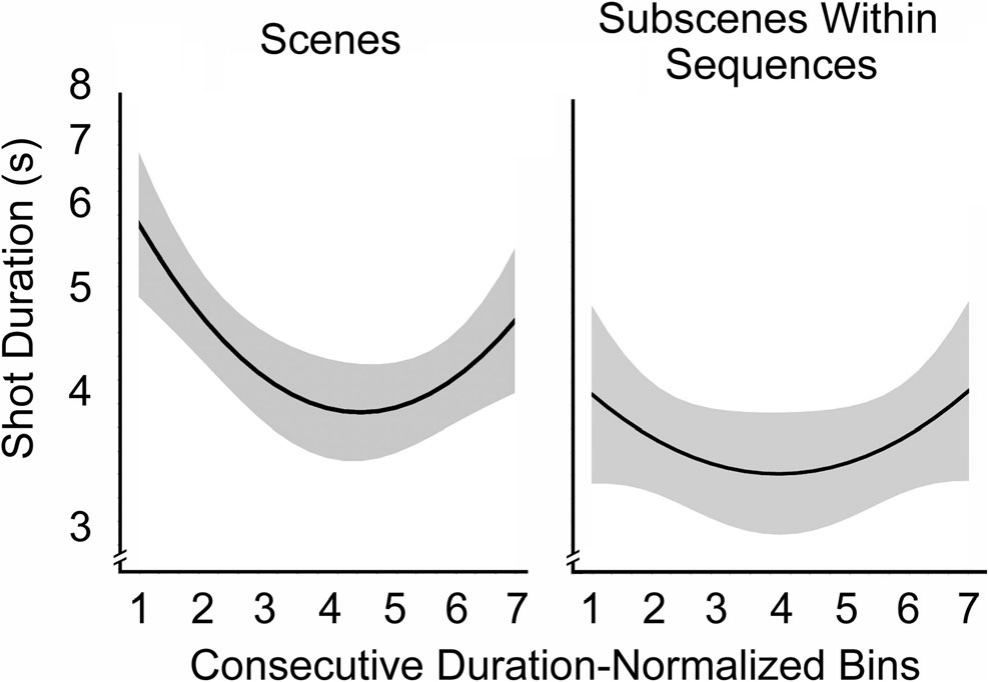
Results of Study 3. The left panel shows the shot-duration structure for scenes, and the right panel that for subscenes within sequences, both with log spacing on the ordinate. These scalloped patterns emerged first by fitting the pattern of shot durations in each of the scenes and subscenes of 20 movies into 200 bins, then averaging within films and across them, and finally resampling those results into seven equally spaced bins, to better approximate independent samples. The bins listed as 1–7 came from Bins 1, 33, 67, 100, 133, 167, and 200 in the earlier sampling. The lightly shaded areas show the 95% confidence intervals on the quadratic regression lines (in black)

More importantly in this context, the fits to the resampled data are shown in Fig. 3. The left panel shows the fit for shot durations within scenes, and the right for shot durations of subscenes within sequences. Three results stand out. In a multilevel regression for scenes and subscenes nested within movies, scenes were generally longer in duration (4.78 s) than subscenes (4.10 s; Wald *p* = .003). Second, there was an interaction of scenes/subscenes with bins (Wald *p* = .036). In particular, the quadratic trend seen in the left panel of Fig. 3 was substantial [*F*(2, 137) = 7.15, *p* = .001], but that for the subscene durations in the right panel was not [*F*(2, 137) = 1.2, *p* = .30]. Third, and most importantly, a slight difference is visible between these trends (*z* = 1.97, *p* = .05). Again this can be seen in Fig. 3, with the scalloped profile for subscenes being shallower and less distinct at the ends than is that for scenes.

Unlike in the results for duration, there was no effect of the shot scales of scenes versus subscenes, and no difference across bins for scenes and subscenes. Unfortunately, there were too few montages (28) across the relevant movies (13) to perform any meaningful analysis. All that can be said about montages is that the shot durations and shot scales were in the same range as those for scenes and for subscenes.

### Conclusion

It appears that, as I suggested earlier, subscenes have somewhat hidden beginnings and ends. That is, shorter and flattened shot duration patterns would seem to provide some account of the diminished consistency of viewers when segmenting subscenes in sequences from the movie stream. Previously, I had also suggested that music is important. Thus, in Study 4 I compared the effects of music in the context of shot durations, shot scale, and other factors.

## Study 4: Effects of music and other factors on viewer segmentations

I have assumed throughout that sequences are generally accompanied by music, but many scenes also have music. Thus, is music a reliable cue for binding sequences? In addition, the results of Study 3 suggest that shorter and flattened shot durations may detract from segmentation. How do the effects of shot durations and scales compare with the efficacy of other information—particularly music, but also motion, luminance, and clutter—as predictors of event boundaries? My expectation was that, in this ensemble of cues, the presence of music, shot durations, and scale would all affect segmentation, and that increased motion might also, since Bordwell (2006) discussed camera motion as one basis for intensified continuity. However, I expected to find no effects of luminance or clutter.

### Method

I assessed six parameters for *all* 3,100 narrative shifts in the 24 movies of the original sample. I asked how well six sources—shot durations (again log transformed because of their extensive skew), shot scale, music, motion, luminance, and clutter—predict viewers’ segmentations, where all three or just two, one, or no observers might segment at a given narrative shift. *Music* was coded 1 if it was present at any boundary shift in any movie, and 0 if none could be heard; *motion* (or better, zero-order motion) was measured by correlating next-adjacent frames within each shot (e.g., 1–3, 2–4, 3–5, etc.), where lower correlations would represent more motion (Cutting et al., 2012); *luminance* was registered by taking the median luminance value (0–255) in every frame and then averaging those values across every frame within each shot (Cutting et al., 2012); and *clutter* was measured by using an edge-finding algorithm and counting up the number of edge pixels as a function the total number of pixels in each frame, sampled from one out of every ten frames in every shot, and then averaged (Cutting & Armstrong, 2016). Given the large amount of data, only results with an alpha value (*α*) of .0001 were considered. The 24 movies were entered as a nominal regression variable.

### Results

Unsurprisingly, given the results of Study 3, shorter shot durations predicted less consistent segmentation performance [*t*(3054) = 11.5, *p* < .0001, *d* = 0.41], as did tighter shot scales (a slight movement toward more close-ups [*t*(3054) = – 10.5, *p* < .0001, *d* = 0.38], which had previously been shown by Cutting et al., 2012). More importantly, the presence of music also predicted viewers’ less consistent segmentations [*t*(3075) = – 6.0, *p* < .0001, *d* = 0.22]. None of the other three variables—motion, luminance, or clutter—or any interactions involving any of the six variables provided any statistical leverage. A model that included only music, shot durations, and shot scales accounted for 26% of the variance in these segmentation data.

The relatively weaker effect of music is a bit surprising. However, it is surely underestimated in this analysis, for two reasons. First, this analysis overlooked the 9% of sequences that were covered only with dialogue or environmental sounds, which were uncoded in the data. Second, and likely more important, music is used for many different reasons in movies (Chion, 1994; Kalinak, 2010), particularly at points of drama within scenes, and covering successive scenes as well as subscenes. In particular, music occurs over 50% of the shots in these movies, and sequences occur throughout only about 18% of the length of these movies.

### Conclusion

Two sources of information here—music and shorter shot durations—would seem to go a considerable distance in accounting for the diminished consistency of viewers when segmenting subscenes in sequences from the movie stream. To be sure, these results are correlational. However, in experimental studies, Shimamura, Cohn-Sheehy, Pogue, and Shimamura (2015) found that the visual information around cuts in popular movies was more difficult to detect when the soundtrack was included. Likewise, Smith and Santacreu (2017) found that cuts themselves were often masked by music. Such results suggest that sound diffuses viewer attention (see Cohen, 1990, 2013) and that it likely reduces the cognitive resources that might otherwise be available to detect low-level features in the narrational stream. Thus, music and the increased homogeneity of shot durations are reasonable candidates for contributing to perceived continuity.

## General discussion

### Sequences, semiotics, and syntagmas

Semiotics is the study of making meaning. There is a rich tradition of semiotic analyses in the study of language (e.g., Eco, 1984; Shapiro, 2008), spreading through all forms of communication. It was inevitable, then, that such analysis should also spread to the study of movies. Christian Metz (1974a, 1974b) was the most important progenitor, projecting his syntagmatic analysis onto film structure. His analyses considered the orders of shots, particularly in scenes and sequences. Metz’s (1974a) system begins by separating syntagmas from the autonomous shot. Many of the latter are long-duration shots that act like scenes. Others are *inserts*, which are single shots injected into normal scenes. The latter type are by far the most common in this sample, but again are single shots, not sequences.

Metz’s (1974a, 1974b) syntagmas emphasize time, first by describing sections of film that are achronological and chronological. The former group includes two types. The first is the *parallel syntagma*, which typically alternates motifs in which the two threads have no commitment to time. These are rare in popular cinema, but an oft-discussed example is the alternation of shots of wealth and poverty,^4^ which are typified by showing different locations, with no central characters or commitment to changes in time: [L – –]. The second type is the *bracket syntagma*, which consists of shots that have thematic but no inherent temporal relation to one another: [– C –]. *Ordinary People* (*Redford, 1980) has one of these, with 15 sequentially unrelated shots of several collections of people at cocktail/birthday party, including main characters.

The chronological group includes five types. One is the *descriptive syntagma*, in which there is locational change across shots at a moment in time but no alternation, no real characters, and no formal intershot commitment to forward progression (Bateman, 2007): [L – –]. For example, *Ordinary People* (*Redford, 1980) also has one of these in its opening montage—a collection of seven shots of autumn images of a suburban setting near Lake Michigan, separated by dissolves.

The other four chronological syntagmas concern the narrative, and these are more central to my analysis. First is the *alternate syntagma*, which goes back and forth between story threads, as I suggested earlier for the protagonists and antagonists in action movies, such as in the car/train chase sequence in *The French Connection* (*Frankenheimer, 1971), or the chase sequence through Mombasa in *Inception* (*Nolan, 2010)—both [L C –]. Next there are the linear syntagmas, the most prominent of which is the *scene*, which, in the context of Metz (1974a), standard film practice, and this discussion, implies both temporal and spatial continuity.

Finally, there are two syntagmas that Metz (1974a) calls *sequences proper*. Both have ellipses of time deviating from an otherwise continuous narrational stream. The *episodic sequence* has been described as an “organized discontinuity” of shots (Monaco, 2000). The classic example is the breakfasting montage in *Citizen Kane* (*Welles, 1941), in which the devolution of Kane’s marriage takes place over years at breakfast, with adoring and then harsh words, and then silence between Kane (Orson Welles) and his wife Mary (Agnes Moorehead): [– – T]. The *ordinary sequence* is a series of shots that proceed in chronological order but with ellipses of time. One ordinary sequence, showing time compression, can be seen in *The Martian* (*Scott, 2015)—[– – T]—with jumps in time as Watney (Matt Damon) off-loads weight from the escape vehicle in hopes that he will be able to return to Earth. To be frank, however, many scholars (e.g., Bateman, 2007; Monaco, 2000) have had difficulty distinguishing episodic from ordinary sequences.

In summary, several Metzian syntagmatic categories are useful in this context. However, from my perspective, his syntagmas and sequences are variants of the same species. Therefore, I have called them all *sequences* and contrast them to *scenes.* Moreover, although each Metzian case can be fit into the scheme I have presented here, many readers have found Metz confusing. My hope is that the system presented here has a clearer basis and can have a broader application to both psychology and film studies.

### Sequences as grist for event segmentation theory

According to event segmentation theory (Zacks & Swallow, 2007, p. 83), perceivers automatically parse the ongoingness of the world around them into events. This process reduces the “continuous flux of activity to a modest number of discrete” mental entities, for the purpose of achieving an “economy of representation.” In the real world as well as in movies, observers can typically give names to these chunks. Space and time are the primary dimensions along which segmentations are made, and these can be tracked by perceptual means. But also important are goals, which in the context of movies are the property of the characters. These are tracked by viewers, in connection with perceptual information, by cognitive means akin to theory of mind.

In addition, while watching ongoing activity in the real world, a perceiver/viewer automatically makes predictions about what will happen next (Zacks et al., 2007). Such predictions can be important for planning appropriate future action, if necessary. Discrepancies between predictions and ongoing activity are critical for the evaluation of the event context. The mismatch creates an error signal in the perceiver and suggests that an old event has terminated and a new event has begun.

The application of event segmentation theory to movies is fairly straightforward. First, scenes are events (Cutting, 2014; Cutting et al., 2012; Magliano & Zacks, 2011; Tan, 2018; Zacks, 2015). The generally correct predictions by the viewers about what will happen next within a scene suggest the absence of a sufficient error signal, which affords further predictions and the updating of the current event model. The means by which filmmakers try to assure the absence of error signals within a scene is largely through the process of continuity editing (Zacks, 2015). Typically, discontinuity occurs at scene boundaries. These are locations where narrative shifts have occurred—changes in location, characters, and/or time.

But a unit not previously investigated in event cognition has been the sequence, perhaps in part because sequences have been ill-defined. The sequence, and aspects of its structure, can be said to lie between the continuity of shots within a scene and the discontinuity of scene boundaries. As defined here, sequences are composed of subscenes, and they have most of the properties of scenes—that is, they are bounded at their beginnings and ends by narrative shifts of generally the same kind and distribution (Study 1 and Table 1). There are seven types of these shifts, populating the possibilities of one or more changes in location, characters, or time. The puzzle that spurred this investigation is that viewers segment movies at subscene boundaries within sequences considerably less often than at scene boundaries (Study 2).

Like other aspects of continuity editing, subscenes within sequences can be said to inhibit the propagation of an error signal through several means (Studies 3 and 4)—through (a) coverage by music or some other auditory signal, (b) shorter shot durations (likewise allied with midscenes), and (c) flatter shot-duration profiles, diminishing the typically scallop-shaped profile of scenes. The argument from event segmentation theory would be that all of these elements reduce the magnitude of the discrepancy signal in a viewer’s ongoing assessment of the narration. Thus, the notion here is that the chance of detecting an error signal during a subscene is not as great as that between two regular scenes, so that segmentation is less frequent.

### Conclusion

Understanding the cognitive processes of a movie viewer is a daunting task. Yet movies, given the general synchrony of the neural processes of most viewers (Baldassano et al., 2017; Hasson, Furman, Clark, Dudai, & Davachi, 2008), provide an opportunity to assess what the mind is doing over the course of about 2 h. Moreover, popular movies drive technology to diversify the quality of experience, and they also capitalize on storytelling, one of the oldest human endeavors. Stories are made up of strings of recounted events. The purpose of this article has been to show that some events in movies are better called *sequences*, and that their study can further elucidate our understanding of both event segmentation and its scope.

Finally, one may ask whether filmmakers are aware of making sequences in films that conform to all of these structural attributes. I have found very little written by filmmakers about sequences that reflects any of the results reported here. I expect the music results would surprise no filmmaker; they have been aware of the many effects of music since well before Eisenstein (1949). Some might also be aware, following Bordwell (2006), of the shorter shot durations within sequences, since sequences are generally faster-paced than scenes. However, I expect that few if any filmmakers are overtly aware of the flattened pattern of shot durations in subscenes. The reason is that filmmaking is a craft; filmmakers know how to make films, but they do not typically care much about any empirical or theoretical notions of the underlying structures they produce. They are busy people, and just want to make good movies.

## Footnotes

1 Cutting (2014) used a different coding system, keeping the order of location, character, and time within brackets, but signaling the presence of changes with a code of 1, and their absence with a code of 0. Thus, [010] indicated a character change without location or time changes. Some readers found this system difficult to remember, so I have changed it here. Thus, [010] is now [– C –].

2 An asterisk, in both the text and the References, indicates that the sequence in question is available on my website under Movie Clips, at http://people.psych.cornell.edu/~jec7/data.htm.

3 In addition, explosions, crashes, and similar extreme events are often presented from several perspectives, showing a single event three times (thus, necessarily backing up a few hundred milliseconds twice). These are called *stutter cuts* or *triple takes*, and they are almost exclusive to action films. I ignore them here, regarding the displayed time as continuous. The same is true for possible backups of two or more frames across action cuts in the same scene (see Shimamura, Cohn-Sheehy, & Shimamura, 2014), and for small, one-time elisions within a scene, such as cutting out a few steps as an office character walks from her desk over to a coworker. The latter is called “removing the shoe leather” from a scene (Bordwell, 2002).

4 Wealth and poverty are often contrasted in video stills in parallel syntagmas. One can be found here: https://www.youtube.com/watch?v=koJEofG4kvY.
